# Combination therapy of Epidermal Growth Factor and Growth Hormone-Releasing Hexapeptide in acute ischemic stroke: a phase I/II non-blinded, randomized clinical trial

**DOI:** 10.3389/fneur.2024.1303402

**Published:** 2024-04-04

**Authors:** Francisco Hernández-Bernal, Donner Estenoz-García, Juan H. Gutiérrez-Ronquillo, Yenima Martín-Bauta, Karen Catasús-Álvarez, Mario Gutiérrez-Castillo, Marbelys Guevara-Rodríguez, Aliuska Castro-Jeréz, Yoandra Fuentes-González, Yulemis Pinto-Cruz, Carmen Valenzuela-Silva, Verena L. Muzio-González, Héctor Pérez-Saad, Nelvys Subirós-Martínez, Gerardo E. Guillén-Nieto, Diana Garcia-del-Barco-Herrera

**Affiliations:** ^1^Clinical Trial Direction, Center for Genetic Engineering and Biotechnology, Havana, Cuba; ^2^Department of Comprehensive General Medicine, Latin American School of Medicine (ELAM), Havana, Cuba; ^3^Neurology Department, “Antonio Luaces” Hospital, Ciego de Ávila, Cuba; ^4^Neurology Department, “Arnaldo Milián” Hospital, Santa Clara, Villa Clara, Cuba; ^5^Neurology Department, “Celia Sánchez” Hospital, Manzanillo, Gramma, Cuba; ^6^Institute of Cybernetics, Mathematics, and Physics, Havana, Cuba; ^7^Neuroprotection Project, Biomedical Research Direction, Center for Genetic Engineering and Biotechnology, Havana, Cuba; ^8^Biomedical Research Direction, Center for Genetic Engineering and Biotechnology, Havana, Cuba; ^9^Department of Physiology, Latin American School of Medicine (ELAM), Havana, Cuba

**Keywords:** ischemic stroke, brain ischemia, epidermal growth factor, growth hormone-releasing hexapeptide, clinical trial, combination drug therapy, neuroprotection, effect size

## Abstract

**Objective:**

This study tested the hypothesis that a neuroprotective combined therapy based on epidermal growth factor (EGF) and growth hormone-releasing hexapeptide (GHRP6) could be safe for acute ischemic stroke patients, admitting up to 30% of serious adverse events (SAE) with proven causality.

**Methods:**

A multi-centric, randomized, open-label, controlled, phase I-II clinical trial with parallel groups was conducted (July 2017 to January 2018). Patients aged 18–80 years with a computed tomography-confirmed ischemic stroke and less than 12 h from the onset of symptoms were randomly assigned to the study groups I (75 μg rEGF + 3.5 mg GHRP6 i.v., n=10), II (75 μg rEGF + 5 mg GHRP6 i.v., n=10), or III (standard care control, n=16). Combined therapy was given BID for 7 days. The primary endpoint was safety over 6 months. Secondary endpoints included neurological (NIHSS) and functional [Barthel index and modified Rankin scale (mRS)] outcomes.

**Results:**

The study population had a mean age of 66 ± 11 years, with 21 men (58.3%), a baseline median NIHSS score of 9 (95% CI: 8–11), and a mean time to treatment of 7.3 ± 2.8 h. Analyses were conducted on an intention-to-treat basis. SAEs were reported in 9 of 16 (56.2%) patients in the control group, 3 of 10 (30%) patients in Group I (odds ratio (OR): 0.33; 95% CI: 0.06–1.78), and 2 of 10 (20%) patients in Group II (OR: 0.19; 95% CI: 0.03–1.22); only two events in one patient in Group I were attributed to the intervention treatment. Compliance with the study hypothesis was greater than 0.90 in each group. Patients treated with EGF + GHRP6 had a favorable neurological and functional evolution at both 90 and 180 days, as evidenced by the inferential analysis of NIHSS, Barthel, and mRS and by their moderate to strong effect size. At 6 months, proportion analysis evidenced a higher survival rate for patients treated with the combined therapy. Ancillary analysis including merged treated groups and utility-weighted mRS also showed a benefit of this combined therapy.

**Conclusion:**

EGF + GHRP6 therapy was safe. The functional benefits of treatment in this study supported a Phase III study.

**Clinical Trial Registration:**

RPCEC00000214 of the Cuban Public Registry of Clinical Trials, Unique identifier: IG/CIGB-845I/IC/1601.

## 1 Introduction

Ischemic stroke remains an important target for novel preventive and therapeutic strategies. It is estimated that 15 million people worldwide are affected by stroke every year, 5 million of them die and another 5 million of them suffer from long-term disability (1). The global population aged 65 years and over is growing faster than all other age groups, with a concomitant increase in stroke incidence (2). Furthermore, stroke among COVID-19 patients is associated with a significant risk of early mortality (3).

Currently, reperfusion therapy with thrombolytic drugs or endovascular thrombectomy represents the only approved therapeutic approach for acute stroke (4, 5). However, these approaches are associated with a narrow therapeutic window, increased risk of hemorrhagic transformation, and the high cost required to deliver these treatments (6), which limit their suitability for patients (~3%−6%), particularly in low-income countries (7). Moreover, despite treatment, recovery may be incomplete in a significant proportion of patients (8), as the vascular dynamics following recanalization do not invariably reduce tissue injury or reverse functional deficits (9, 10), and there remains scope for additional pharmacological-based neuroprotective interventions in addition to recanalization in acute ischemic stroke.

Considering the strong interdependence of elements in the neurovascular unit (11) and the limitations of neuron-protective strategies in clinical trials (12, 13), the scientific community has moved to combination strategies that seek to enhance endogenous mechanisms of neuroprotection (14). Combined therapies for stroke are likely to be more effective as they simultaneously target multiple levels of the ischemic pathophysiological cascade. However, few clinical trials have been published to date (2).

Molecules that trigger cytoprotective effects can be considered candidates for combined therapies (8). Particularly, a combined therapy based on epidermal growth factor (EGF) and growth hormone-releasing hexapeptide (GHRP6) has demonstrated benefit in preclinical contexts by activating pleiotropic endogenous mechanisms of survival and brain protection (15–19). Both molecules cross the blood-brain barrier (20–23), and their receptors are widely distributed in brain tissues (24, 25). EGF and GHRP6 share common properties such as anti-apoptotic (26, 27) and anti-excitotoxic effects (28, 29). In addition, both molecules have independent biological effects. Specifically, EGF promotes neurogenesis and remyelination (30), while GHRP6 induces endogenous neuroprotective factors such as growth hormone and insulin, like growth factor 1 (31).

Previous results of our group demonstrated the therapeutic benefits of this combination in animal models of multiple sclerosis (15), proximal axonopathy mimicking ALS, and focal and global ischemic stroke (16). Later, EGF+GHRP6 combined therapy improved both clinical and pathological aspects as it reduced neurological symptoms and brain infarct volume, preserving neuronal density (17). Additionally, EGF+GHRP6 combined therapy achieved similar results in a preclinical context when compared to therapeutic hypothermia (18). Moreover, both active ingredients exhibited a high safety profile in preclinical and clinical trials (32, 33).

Supported by these data, a phase I/II randomized clinical trial was designed to test the hypothesis that administration of a combined therapy based on rEGF and GHRP6 at two dose levels is safe for acute ischemic stroke (AIS) patients, admitting up to 30% of serious adverse events (SAE) in relation to proven causality. The therapeutic effect was assessed as a secondary endpoint. The current report has been written in compliance with the Consolidated Standards of Reporting Trials (CONSORT) guidelines for randomized controlled trials (34, 35).

## 2 Patients and methods

### 2.1 Drugs and reagents

Growth hormone-releasing hexapeptide (GHRP6) (His-d-Trp-Ala-Trp-d-Phe-Lys-NH_2_) was synthesized by BCN peptide (Barcelona, Spain). *Saccharomyces cerevisiae* is the host organism that expresses recombinant epidermal growth factor (rEGF). Both ingredients were formulated separately in lyophilized preparation for intravenous administration at the facilities of the Center for Genetic Engineering and Biotechnology (Havana, Cuba).

EGF and GHRP6 were retained at 2–8°C at all times to guarantee the cold chain. Packages containing 14 kits, corresponding to the 14 administrations for each patient, were sent to the pharmacy of each clinical site. Trained personnel, under researcher supervision, carried out the preparation and administration of the study drugs.

### 2.2 Ethics approval and consent to participate

This study was conducted according to the ethical principles of protection of participants in biomedical research stated in the Guidelines of Good Clinical Practice (CECMED 2000, Cuba) (36), the Guide of Good Clinical Practices of the International Conference on Harmonization (ICH E6) (37), and the Declaration of Helsinki (38).

The study protocol (Code: IG/CIGB-845I/IC/1601) was approved by the Ethics and Review Committee of every hospital involved in this trial and by the Cuban Center for State Control of Drugs, Medical Devices, and Equipment (CECMED, in Spanish). The Ethics and Review Committees also supervised the study execution, ensuring the protection of the rights, safety, and well-being of the participants involved in the study and verifying the progress of the clinical trial and the investigators' adherence to the protocol. This clinical trial was also registered at the Cuban Clinical Trial Public Registry No. RPCEC00000214 (https://rpcec.sld.cu/en/trials/RPCEC00000214-En).

Verbal and written informed consent was obtained from all patients or their legally authorized representatives regarding interventions, clinical assessments, and all features related to the current research (see below).

### 2.3 Trial design

This was a multicenter, open-label, controlled, phase I/II clinical trial with centralized randomization and parallel groups. The primary endpoint was the assessment of the safety of two dose levels of EGF+GHRP6 combined therapy in patients with acute ischemic stroke, in addition to secondary endpoints of therapeutic effects.

The trial was performed in stroke units of 11 hospitals from eight provinces of Cuba from July 2017 to January 2018. The participating researchers were internists, neurologists, and emergency room physicians.

The sample size was estimated according to a specific method for pilot studies of transition therapies to estimate an upper limit to the related serious adverse event rate of 30% (39). Assuming a type I error of 0.05, a sample size of 10 subjects per group was estimated.

**Figure 1:**
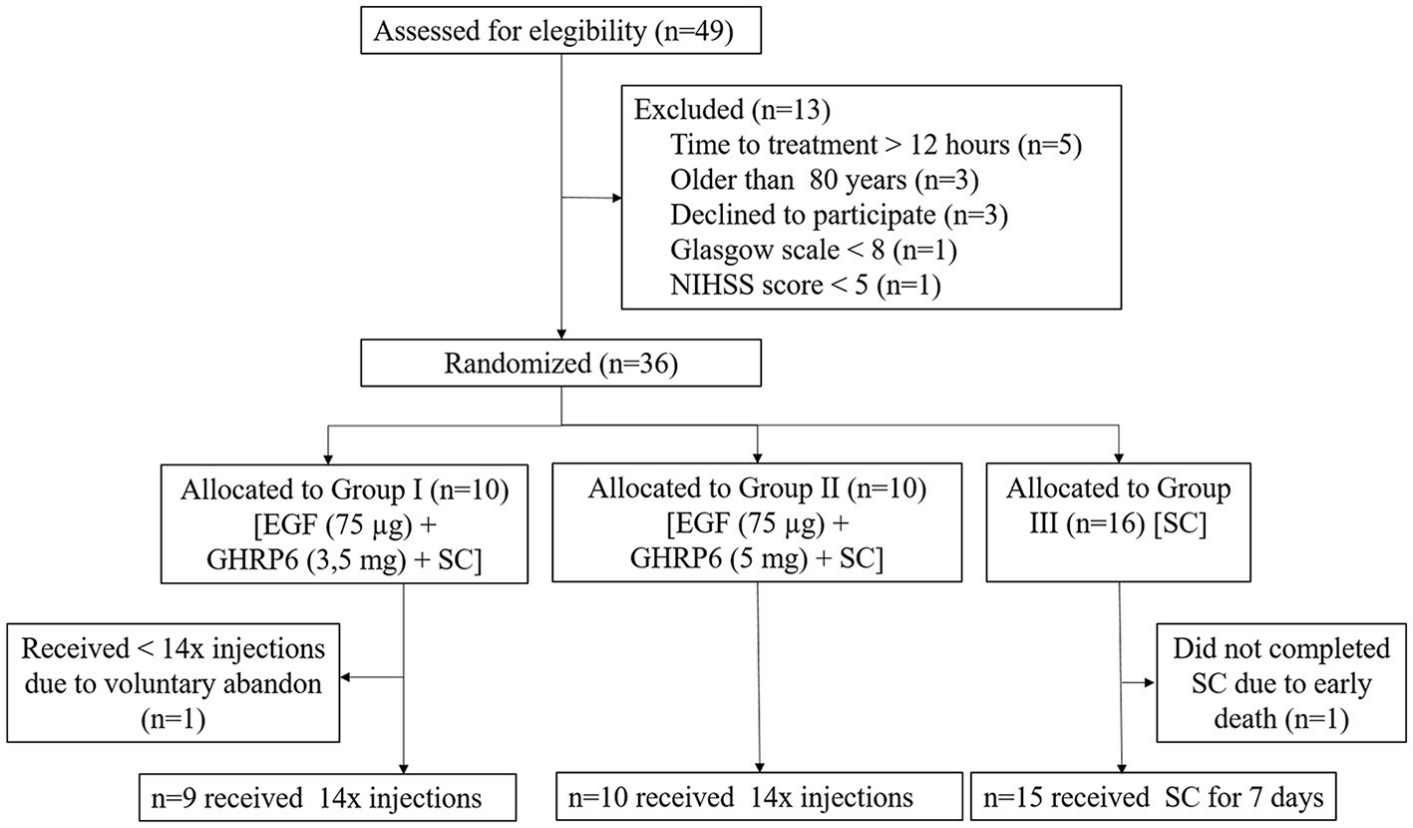
CONSORT flow diagram of the study. Allocation of patients and causes of non-compliance. A total of 36 patients were randomized and included in the final analysis. SC, standard care.

However, a protocol deviation occurred at the beginning of the study because a delay in enrollment and treatment procedures led to unmet therapeutic window criteria for the first six patients. Thus, it was decided to include them in the control group. On completion of the study, a total of 36 patients were included (Figure 1).

The randomization procedure was carried out through a randomized, centralized list based on three-subject blocks using the “2N” computer tool (University of Arkansas). After verbal consent was obtained, clinician researchers enrolled patients without previous knowledge of their group assignment and then phoned the central trial coordinator (HBF), who assigned the identification code and the study group. Subsequently, the study drugs were prescribed accordingly and requested at the hospital's pharmacy.

Patients assigned to the control group (n = 16) only received standard care for stroke in the acute phase, which did not include recanalization (40). Two other groups were treated with standard care plus EGF 75 μg + GHRP6 3.5 mg (Group I, n=10) or EGF 75 μg + GHRP6 5 mg (Group II, n=10). Dose selection was based on previous preclinical and clinical studies and two methods of dose extrapolation from rodents to humans, which considered body surface and body weight (41). Both substances were slowly injected intravenously twice a day, 12 h apart, and over the course of 7 days, with EGF being administered first.

Standard care for all groups included symptomatic control of comorbidities such as hypertension and diabetes. Patient stabilization, including airways, breathing, and circulation, was achieved through continued vital sign monitoring, hydration, and adequate oxygenation, among other measures included in the Cuban National Program of Care for patients with cerebrovascular disease (40). In the case of those with impaired consciousness or a large mass effect with midline displacement in the CT scan, 20% mannitol (0.25–0.5 g/kg) was administered every 4 h, and hyperventilation therapy was considered. Any concomitant medication was delivered according to the medical criteria. Recanalization interventions were not applied.

Treatment was completed in the hospital admission regime. Hospital discharge occurred at least 12–24 h after the last administration of the combined therapy.

### 2.4 Inclusion criteria

Eligible participants were adults aged 18–80 years with a focal acute neurological defect caused by an acute ischemic stroke (a hemorrhagic event was excluded by computed tomography), with less than 12 h between the onset of symptoms and therapy initiation. Verbal consent was obtained from the patient or their legal representative. Once patients were stabilized, in the following days, they were asked for a written and signed consent form to continue participating.

### 2.5 Exclusion criteria

Exclusion criteria were coma state (Glasgow scale less than 8), NIHSS scale score < 5 or > 20, a neurological defect explainable by a condition other than ischemic stroke, patients with quickly resolving neurological symptoms, severe and uncontrolled arterial hypertension (systolic > 185 mmHg or diastolic > 110 mmHg) or arterial hypotension (systolic < 95 mmHg) both unresponsive to standard treatment, seizures, and patients with a diagnosis of malignant neoplasms, pregnancy, puerperium, or mental disorders.

### 2.6 Stopping rules

If a related serious adverse event rate higher than that allowed in the hypothesis (30%) with a high probability (> 0.90) occurred in the highest dose group of GHRP6 (5.0 mg), inclusion and treatment would be stopped in that specific group. If this phenomenon occurred in Group I (3.5 mg GHRP6), the clinical trial would be stopped. Safety and primary data were assessed and reviewed by an independent data monitoring committee consisting of a statistician, a clinician, an epidemiologist, and a neurologist. None of the treatment groups in the present study were closed.

### 2.7 Primary and secondary endpoints

As a phase I/II clinical trial, the primary endpoint was the safety of intravenously administered EGF+GHRP6 combined therapy in the setting of an acute stroke. Clinical adverse events [type, intensity, evolutionary outcome, and causal relationship (42)], vital signs, and electrocardiographic monitoring were recorded during the 7-day in-hospital stay and in outpatient visits at 1, 3, and 6 months after discharge. Patients were instructed to observe adverse events during the study period and report them to their attending physician.

Combined therapy was considered safe if not associated with an adverse event degree ≥ 3. The treating physicians determined causality at the time of the event based on their expertise and previously reported adverse events for each independent component, which was further reviewed by the data monitoring committee. Considering that both EGF and GHRP6 had previous clinical studies focused on other conditions, the expected adverse effects were mainly fever, shivering, and vomiting for the former (43), and sweating and bradycardia for the latter (32). The causes of death were registered based on clinical evidence and necropsy findings.

Secondary endpoints included neurological (National Institutes of Health Stroke Scale, NIHSS) and functional (modified Rankin scale (mRS) and Barthel index) outcomes at discharge, 3 and 6 months later, and survival 6 months after treatment. At the time of enrollment, patients were evaluated according to the NIHSS (44) to register baseline stroke severity. This scale assesses the degree of neurological deficit in 11 categories, wherein a normal function level without neurological deficit has a zero score and the maximum score is 42 points.

mRS, which is a simplified evaluation of functionality, was used at discharge and during the follow-up period (3 and 6 months). mRS values range from 0 (to indicate no residual symptoms) to 6 (to indicate death). The Barthel Index ranged from 0 to 100, wherein a 100 score indicates no deficit and a 0 score indicates complete dependence or death. Based on these assessments, ordinal and dichotomous data analyses were conducted. mRS ≤ 2 and Barthel index ≥ 85 were considered favorable outcomes (45, 46).

Hematological and biochemical assays were carried out at the beginning of the study, 72 h at hospital discharge, and 1, 3, and 6 months later. The Isotope Center (CENTIS) laboratories performed hormonal assays (for growth hormone (hGH), ACTH, aldosterone, cortisol, prolactin, and insulin), for which sera were collected at the beginning of the study, 72 h, at hospital discharge, and 1, 3, and 6 months later.

### 2.8 Quality assurance

Before study initiation, a workshop took place to unify criteria with researchers involved in this trial. Trained personnel carried out visits and quality monitoring in 100% of the hospitals participating in the study to verify compliance with the Good Clinical Practices.

### 2.9 Data handling and statistical analysis

The primary objective was to establish that the intervention was safe, admitting up to 30% of serious adverse events with a proven causal relationship.

For the purposes of this study, a data entry system was created in OpenClinica software (www.openclinica.com), with Data Collecting Notebook (DCN) as a primary data source. After review and query resolution, information was double-entered for subsequent automatic comparisons and corrections until no differences between databases were found. After completion, databases were closed and exported for the corresponding statistical analyses; no modification of primary data was allowed. SPSS software version 26.0 and EPIDAT v. 4.1 were used for statistical analyses. All analyses were done on an intention-to-treat (ITT) basis.

Adverse events were reported as descriptive statistics. Recurrent events were counted as separate events. The effect size was calculated based on the odds ratio (OR) with a 95% CI. The probability of treatment-related serious adverse events was evaluated iteratively with a Bayesian algorithm, as well as the criterion for stopping due to an unacceptable related serious adverse event rate with Beta (0.5, 0.5) as *a priori* distribution (47).

Categorical data were compared using the χ^2^ and Fisher's exact tests. For quantitative variables, the mean and standard deviation, minimum and maximum values, and confidence intervals for the mean were determined. For group differences, ANOVA or the Kruskal-Wallis tests were used according to the variable's nature. In the cases of normal approximation, groups were compared using the Bayesian method for independent samples by means of the Bayes factor, assuming diffuse priors for unequal variance between groups (Rouder method) (48). When applicable, the effect size was reported as the mean difference with a 95% CI accompanied by the *Hedges' g* (49, 50). Deceased patients were given the highest possible score on the previously mentioned scales.

Survival of both treated groups vs. control was analyzed using a log-rank (Mantel-Cox) test and a Cox regression model using the hazard ratio and 95% CI as effect sizes.

Ancillary analyses included the ordinal assessment of mRS and the survival of merged treated groups. Spearman's correlation coefficients were calculated in order to assess the likeness between the EGF+GHRP6-treated groups. In addition, utility-weighted mRS (UW-mRS) (51) and quality-adjusted life-years (QALY) gained by the intervention were calculated (52). For UW-mRS data, the utility weights proposed by Chaisinanunkul N. et al. (51) (mRS 0–1.0; mRS 1–0.91; mRS 2–0.76; mRS 3–0.65; mRS 4–0.33; mRS 5–0; mRS 6–0) were adopted. To determine the exact QALYs, the utility value associated with a given state of health was multiplied by the specific time (3 and 6 months) lived in that state (52).

## 3 Results

Of the 49 stroke patients screened for participation in the trial, 73.5% (n=36) fulfilled the inclusion criteria. The most common reasons for exclusion were time to treatment longer than 12 h, older than 80 years, and patients' will. In the present study, there was no closure of any treatment group.

### 3.1 Demographic and clinical characteristics

A total of 36 patients were included in the study (Figure 1). One out of 36 patients (from Group I) voluntarily withdrew from the trial after receiving 7 of 14 scheduled injections. This withdrawal was not associated with any risk concerns.

The mean time elapsed from the onset of symptoms to the start of treatment was 7.3 ± 2.8 h overall. No ECG changes were noted in more than 50% of patients in each group. Acute cerebral infarcts had a CT scan-confirmed ischemic etiology at baseline in 100% of cases. Two patients had subsequent hemorrhagic transformations (one patient in Group I with a 6-month survival and one deceased patient in Group III). The most frequent infarct locations were the left parietal (11.1%), left temporal (8.3%), and left temporoparietal (8.3%) zones.

Demographic and baseline characteristics were similar between groups. The mean age was 66 ± 11 years, and 21 were men (58.3%). Other clinical characteristics were also similar between groups, based on comparable grades of infarct symptoms and signs according to the NIHSS scale (Table 1).

**Table 1:** Baseline characteristics of the patients.

**Parameters**		**Group I (EGF_75μ*g*_ + GHRP6_3,5mg_)**	**Group II (EGF_75μ*g*_ + GHRP6_5mg_)**	**Group III (standard care)**
Age (mean ± SD)		69.7 ± 9.4	67.7 ± 11.4	63.5 ± 12
Body mass index (mean ± SD)		27.7 ± 3.3	26.8 ± 2.1	26.3 ± 4.8
Sex (female) *N* (%)		3 (30%)	4 (40%)	8 (50%)
Skin color *N* (%)	White	8 (80%)	4 (40%)	8 (50%)
	Black	2 (20%)	4 (40%)	3 (31%)
	Mixed	0	2 (20%)	5 (19%)
Hypertension *N* (%)		8 (80%)	9 (90%)	11 (69%)
Diabetes *N* (%)		3 (30%)	1 (10%)	7 (44%)
Coronary disease *N* (%)		0	1 (10%)	3 (31 %)
Smoking *N* (%)		1 (10%)	0	5 (19%)
Alcoholism *N* (%)		2 (20%)	1 (10%)	2 (12%)
Time to treatment (h) mean ± SD		9.0 ± 3.1	7.9 ± 2.1	5.9 ± 2.4
Glycemia (mmol/L) mean ± SD		7.4 ± 5	6.7 ± 1.5	6.7 ± 2.6
NIHSS at enrollment (median, 25%−75% interquartile range)		8.5 (5.75–11.25)	8.5 (6.50–13.75)	9 (6–12.75)
Cortisol (nmol/L) mean ± SD		451.9 ± 164.2	624.3 ± 641	419.3 ± 165.7

In general, the laboratory parameters evaluated over time remained within the normal ranges; any variations had no clinical relevance. The hormonal profiles, in all cases, were similar between the three groups and were within the reference values for each hormone (data not shown).

### 3.2 Harms or unintended effects (primary endpoint)

The frequency of adverse events (AE) and serious AEs (SAEs) in the ITT population was similar among all groups (Table 2). In total, 23% of AE (in three and four patients in Groups I and II, respectively) had a probable or definite causal relationship with the intervention therapy. The intensity of these AEs was mainly mild or moderate (Table 3). Additionally, these AEs were completely solved.

**Table 2:** Frequency of patients with serious and total adverse events.

**Adverse events (AE)**	Group I (*n =* 10) *N* (%)^*^	**Odds ratio (vs. GIII) (95% CI)**	Group II (*n =* 10) *N* (%)^*^	**Odds ratio (vs. GIII) (95% CI)**	Group III (*n =* 16) *N* (%)^*^
Overall AE	6 (60)	0.35 (0.06–2.06)	9 (90)	2.08 (0.18–23.30)	13 (81)
AE attributable to therapy	3 (30)	15.4^**^ (0.70–337.23)	4 (40)	22.85^**^ (1.07–487.02)	0 (0)
Overall serious AE	3 (30)	0.33 (0.06–1.78)	2 (20)	0.19 (0.03–1.22)	9 (56)
Serious AE attributable to therapy	1 (10)	5.21^**^ (0.19–141.09)	0 (0)	1.57^**^ (0.03–85.42)	0 (0)

* Data represent patients with at least one episode of adverse event during the study.

** 0.5 is added to all cells (because zeros cause problems with the computation of the odds ratio).

**Table 3:** Relevant expected adverse events in this study.

**Adverse event**		**Group I n_AE_ = 28 (*n*, %)**	**Group II n_AE_ = 30 (*n*, %)**	**Group III n_AE_ = 37 (*n*, %)**
Fever		5 (18)	5 (17)	7 (19)
Intensity (*n*)	Mild	3	2	5
	Moderate	2	3	2
Tremor		3 (11)	-	-
Intensity (*n*)	Mild	3	-	-
Vomiting		2 (7)	3 (10)	-
Intensity (*n*)	Mild	-	1	-
	Moderate	1	2	-
	Severe	1	-	-
Nausea		3 (11)	3 (10)	-
Intensity (*n*)	Mild	1	3	-
	Moderate	2	-	-
Dizziness		-	2 (7)	-
Intensity (*n*)	Mild	-	2	-
Sweating		1 (4)	4 (13)	-
Intensity (*n*)	Mild	1	4	-
Shivering		5 (18)	3 (10)	-
Intensity (*n*)	Mild	2	3	-
	Moderate	3	-	-
Headache		-	3 (10)	3 (8)
Intensity (*n*)	Mild		3	3
Dyspnea		1 (4)	-	-
Intensity (*n*)	Severe	1	-	-
Asthenia		-	2 (7)	1 (3)
Intensity (*n*)	Mild	-	2	1
Epigastralgia		1 (4)	-	-
Intensity (*n*)	Moderate	1	-	-
Bradycardia		-	-	1 (3)

n_*AE*_ refers to the total AEs reported in each group.

A total of 19 serious AEs were reported in 14 of 36 patients (three, two, and nine patients in Groups I, II, and III, respectively; see Table 2). In terms of causality with the interventional drug, only two serious AEs occurred in one patient in Group I (vomiting and dyspnea). In Group II, neither of the two SAEs (respiratory infection and brain reinfarction) reported (leading to death in both cases) had a causal relationship with the therapy under study. A higher probability of related AEs is detected in Group II with respect to Group III (lower limit of 95% CI for OR>1). No other significant association was detected (Table 2). The probability of confirming the study hypothesis (a limit to related serious adverse event rates lower than 30%) was greater than 0.90 in each group (globally greater than 0.99), with a related serious adverse event rate expected between 0 and 0.18 (95% CI).

The most relevant AEs with any causal relationship to investigational drugs are listed in Table 3. Bradycardia, which was one of the expected AEs associated with GHRP6, was not reported in any EGF+GHRP6-treated patients.

A total of seven deaths in Group III (control) and two deaths in Group II were reported. The lowest-dose group reported no mortality. Causes of mortality in Group III were arterial hypotension (1), bronchopneumonia (2), pulmonary embolism (1), acute pulmonary edema (1), respiratory distress (1), and brain edema (1). Deaths in Group II were due to respiratory infection and brain reinfarction. These were not associated with the interventional drugs.

### 3.3 Neurological and functional outcomes

#### 3.3.1 Neurological outcome at discharge and after 3 and 6 months

**Figure 2:**
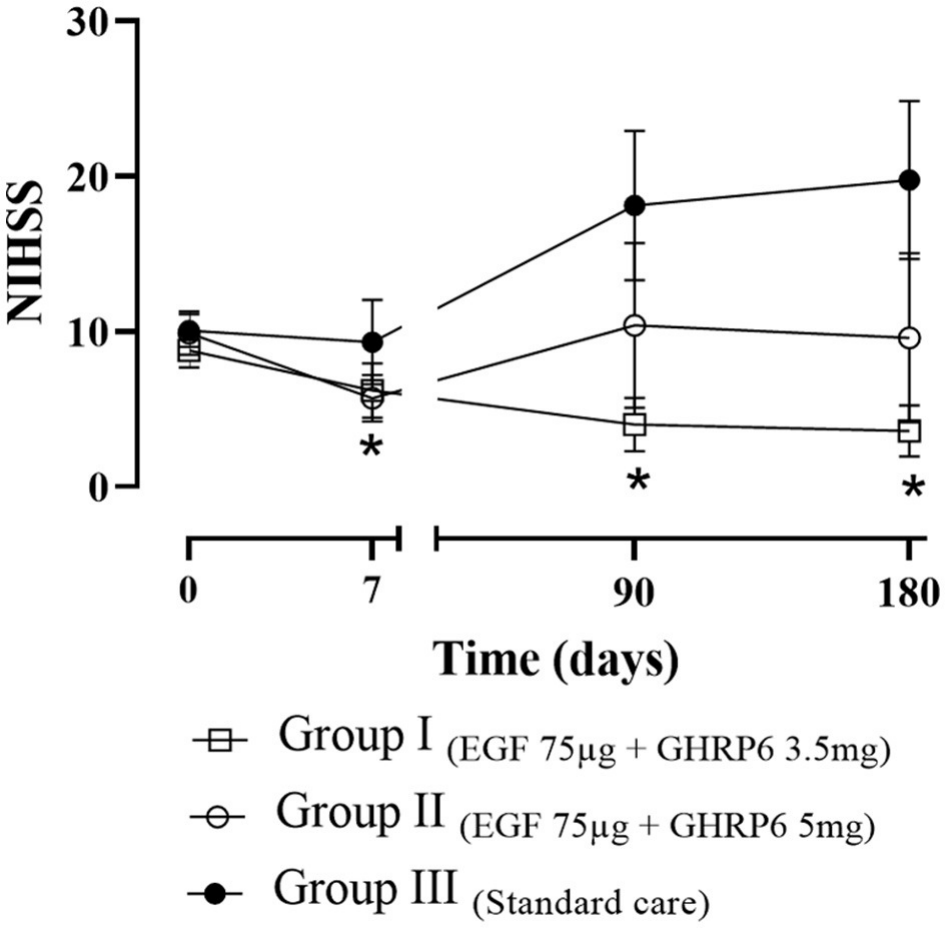
Functional recovery of patients assessed by NIHSS. The data depict the mean ± SEM. Bayesian analysis: * indicates differences vs. the baseline score. Dead patients receive the worst possible score.

The increased stroke severity over time in the control group, observed in Figure 2, was consistent with the worst possible score assigned to dead patients. This fact was influenced equally in all groups and in both neurological and functional assessment tools.

ITT analysis showed a reduction in neurological deficit at 90 and 180 days in patients receiving EGF 75 μg + GHRP6 3.5 mg (Group I) compared to baseline NIHSS score. This reduction is also observed regarding Group III at the same assessment times (probabilities of difference greater than 0.95). In Group II, a reduction in the NIHSS score was recorded only at discharge compared to baseline (Figure 2).

As a measure of changes over the entire period, the areas under the individual curves (AUC) were calculated. The Bayes Factor_1 − 0_ in favor of the difference between Group I and Group III was 2.14 (*p* = 0.031), indicating sufficient evidence to reject the equality hypothesis.

#### 3.3.2 Functional outcome at discharge and after 3 and 6 months

**Figure 3:**
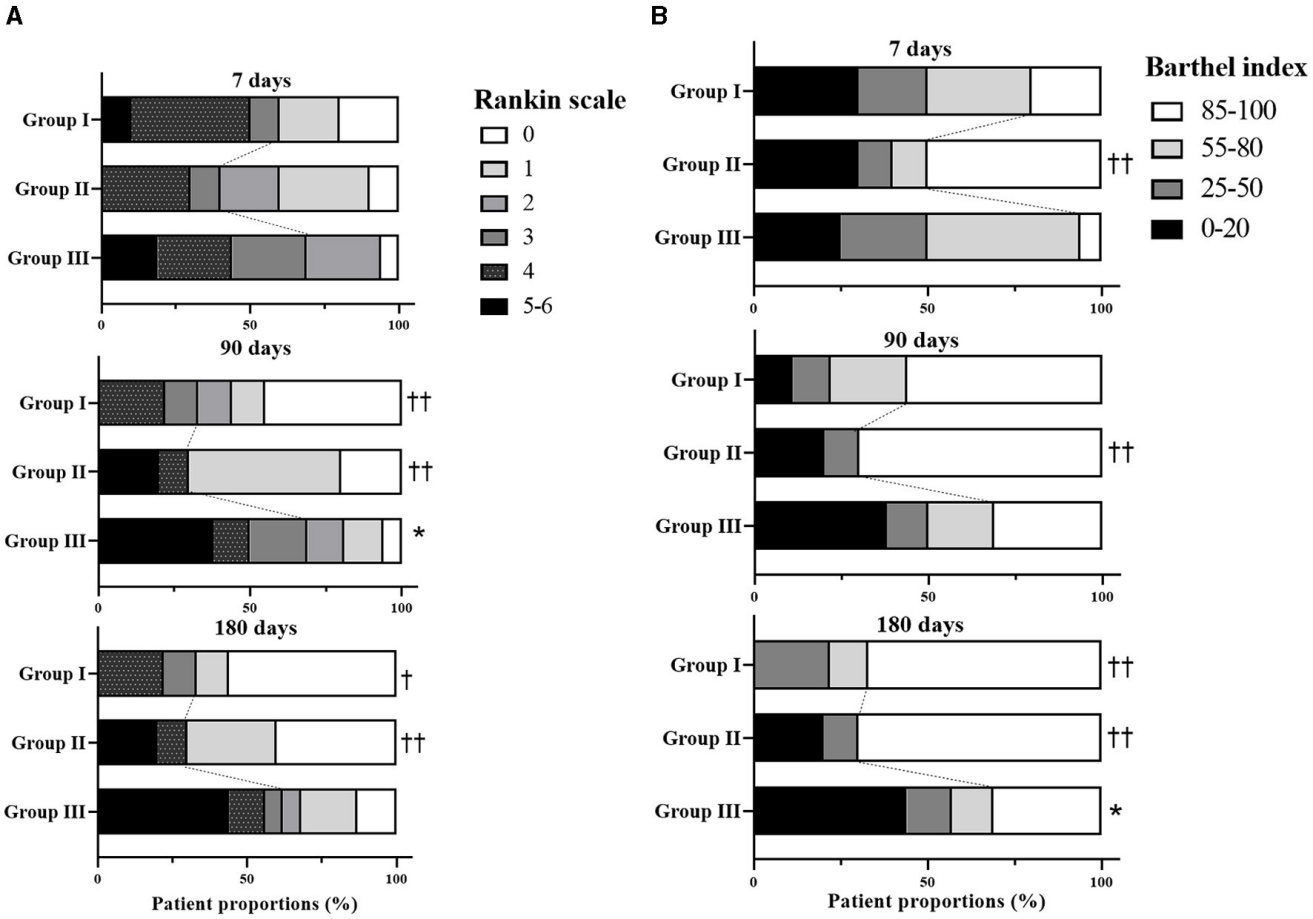
Clinical outcomes in the ITT population: modified Rankin scale (A) and Barthel index (B) at discharge (7 days), 3 and 6 months after stroke onset. * indicates significant differences between Group III and the EGF+GHRP6-treated groups, according to χ^2^ test (*p* < 0.05). ^††^indicates probabilities of difference with respect to Group III >0.95; ^†^probabilities of difference >0.90, based on Bayesian analysis. Dashed lines demarcate the boundaries between the strata considered favorable (0–2 for the Rankin scale and ≥ 85 for the Barthel index) and not favorable. Dead patients received the worst possible score.

Dichotomized analyses of the mRS scale and Barthel index showed an improved functional outcome in EGF+GHRP6-treated groups vs. Group III (control) at 90 days and 180 days, respectively. Notably, this benefit was seen at both ends of the scale (Figure 3).

Effect size analysis based on mean difference and *Hedges' g* for NIHSS and mRS scores showed a strong effect in Group I from 90 to 180 days. Barthel index analysis indicated a moderate effect in both EGF+GHRP6-treated groups (Table 4).

**Table 4:** Summary of the effect size of neurological and functional outcomes.

**Endpoint**	**Group I (*n =* 10) (±SD)**	**Mean difference (95% CI)**	**g*_*Hedge*_***	**Group II (*n =* 10) (±SD)**	**Mean difference (95% CI)**	**g*_*Hedge*_***	**Group III (*n =* 16) (±SD)**
**NIHSS score**							
7 days	6.2 ± 5.6	3.11 (-4.64; 10.9)	0.32	5.7 ± 4.8	3.61 (-4.0; 11.2)	0.38	9.3 ± 11.0
90 days	4.0 ± 5.5	14.1^*^ (1.17; 27.1)	0.88^£^	10.4 ± 16.8	7.72 (-7.54; 23.0)	0.41^§^	18.1 ± 19.2
180 days	3.6 ± 5.2	16.1^*^ (2.51; 29.8)	0.95^£^	9.6 ± 17.2	10.2 (-5.85; 26.1)	0.51^§^	19.8 ± 20.3
**Barthel index**							
7 days	52.5 ± 36.9	10.0 (16.6; 36.6)	0.30	63.5 ± 37.9	21.0 (5.96; 48.0)	0.63^§^	42.5 ± 28.6
90 days	70.5 ± 37.4	23.9 (9.4; 57.2)	0.58^§^	71.5 ± 42.5	24.9 (9.9; 59.8)	0.58^§^	46.6 ± 41.5
180 days	74.5 ± 36.9	27.6 (7.8; 63.0)	0.63^§^	73.5 ± 43.7	26.6 (10.7; 64.0)	0.57^§^	46.9 ± 45.6
**mRS**							
7 days	2.5 ± 1.8	0.8 (0.6; 2.1)	0.45^§^	2.2 ± 1.5	1.1 (-0.2; 2.3)	0.69^§^	3.3 ± 1.5
90 days	1.8 ± 1.8	1.9^*^ (0.2; 3.6)	0.91^£^	2.1 ± 2.3	1.6 (-0.2; 3.4)	0.70^§^	3.7 ± 2.1
180 days	1.6 ± 1.9	2.0^*^ (0.2; 3.9)	0.87^£^	1.9 ± 2.5	1.7 (-0.3; 3.8)	0.68^§^	3.6 ± 2.4

Mean differences were calculated with respect to Group III. ^*^indicates a stronger effect size. ^£^indicates a strong effect (>0.8) and ^§^indicates a moderate effect (~0.5), according to Chhatbar et al. (50).

### 3.4 Survival

**Figure 4:**
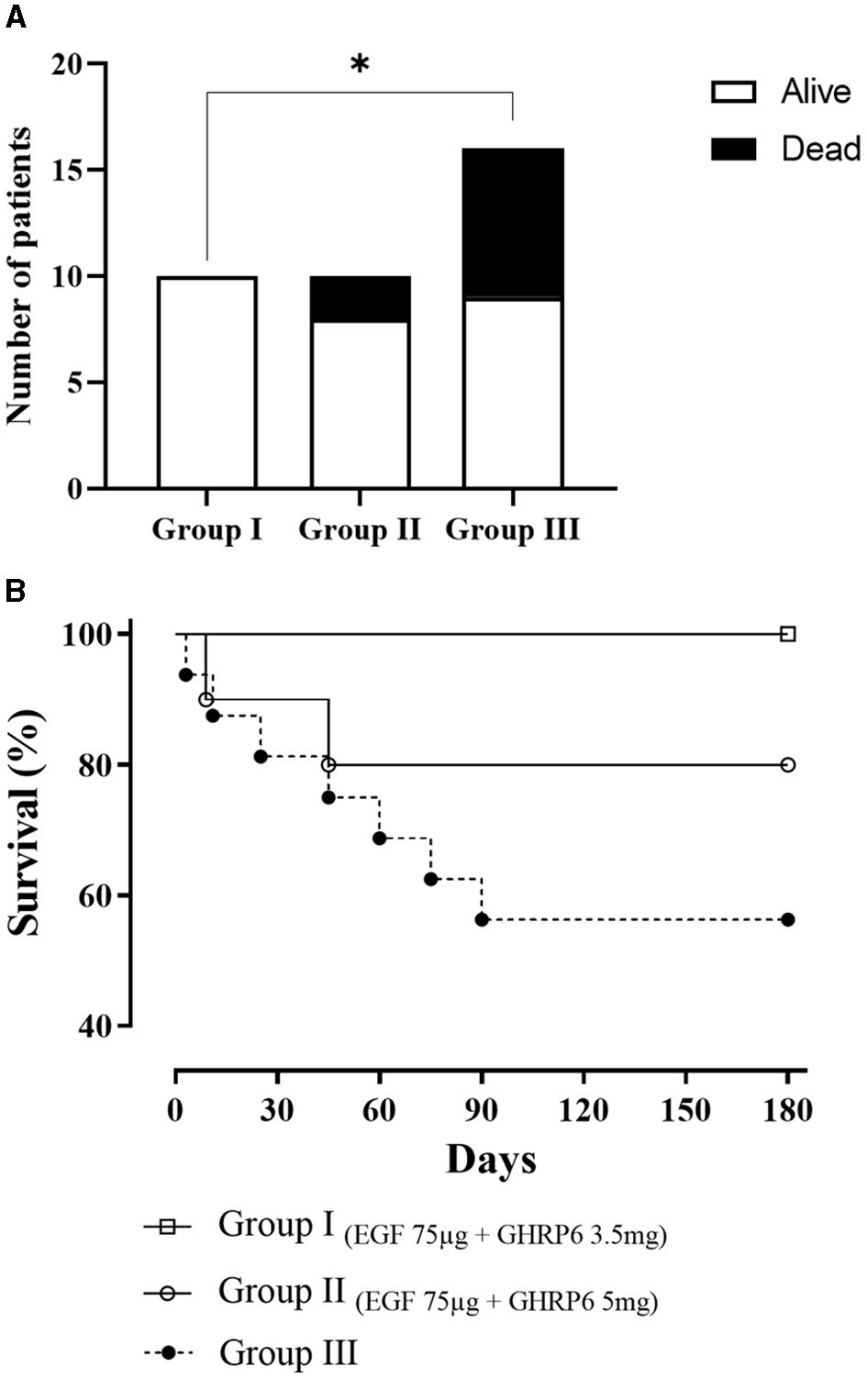
Survival of experimental groups analyzed by **(A**) χ^2^ test at 6 months (*p = 0.03) and **(B)** Kaplan-Meier curves and log-rank test (*p* = 0.053).

At 6 months, proportion analysis evidenced a higher survival of patients treated with the combined therapy (Figure 4A). At this time, Group I treated with EGF 75 μg + GHRP6 3.5 mg showed the highest overall survival (HR=0; *p* = 0.02). Group II (EGF 75 μg + GHRP6 5 mg) achieved an HR of 0.42 (95% CI: 0.111–1.597) at the end of the study. However, the log-rank test showed no statistical differences (Figure 4B).

### 3.5 Ancillary analysis

An analysis of weight-based dosing revealed significant differences between the mean dosages of both groups (*p* < 0.0001, t-test). Additionally, the relationship between dosing and mRS showed a probability of differences between Group I and Group II less than 0.6, with symmetrical confidence intervals around 0, suggesting similarity between both treated groups (data not shown).

**Figure 5:**
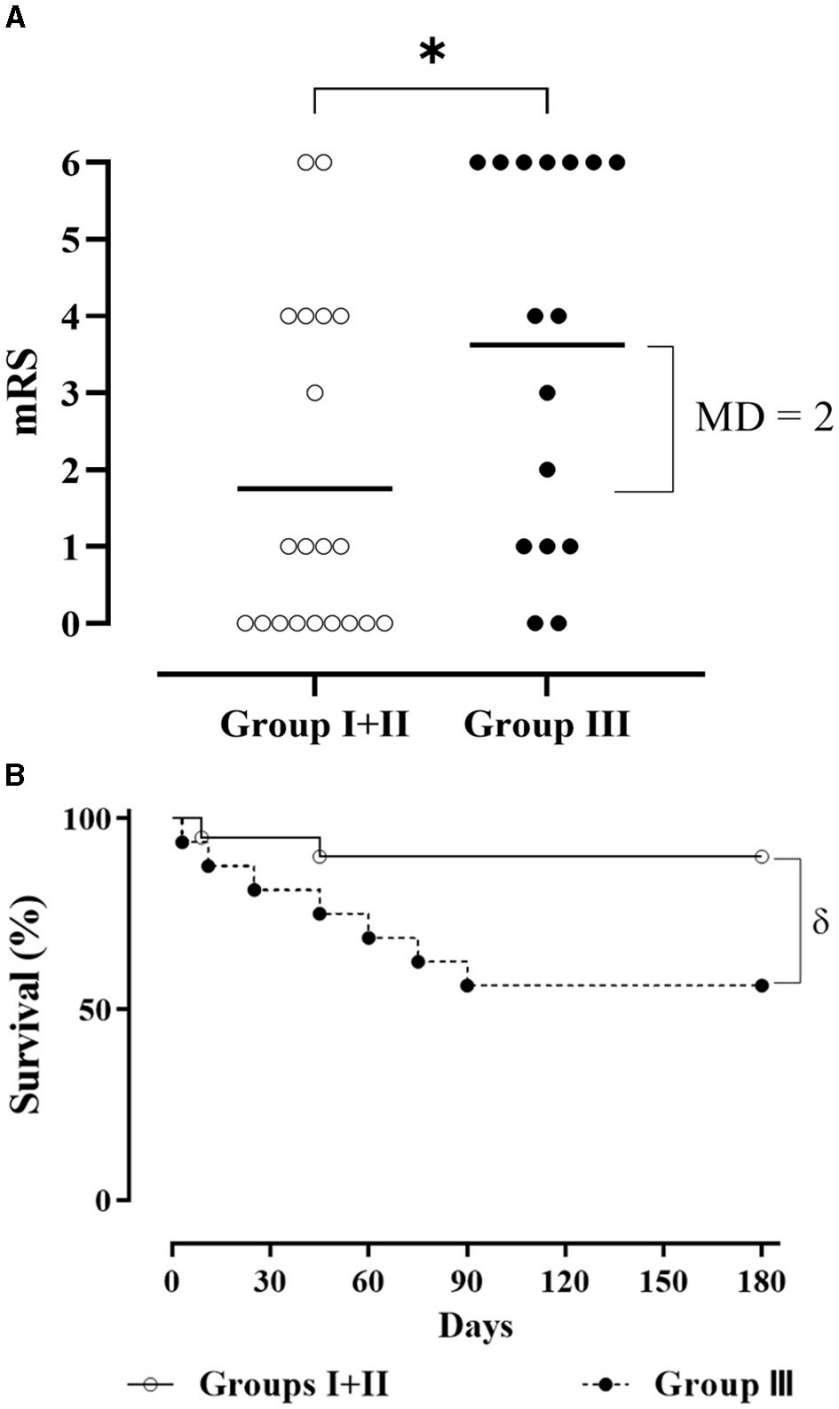
**(A)** mRS score of EGF+GHRP6-treated patients (Groups I and II; empty circle) and Group III (filled circle) at 180 days (^*^: *p* = 0.015, according to the Mann-Whitney U test). MD, mean difference. **(B)** Survival Kaplan-Meier curves in groups analyzed [δ: *p* = 0.024, HR 0.21 (95% CI: 0.06–0.82) according to log-rank test].

Thus, for an additional purpose in terms of therapeutic effects, an ordinal analysis of mRS was conducted, in which merged treated groups showed a significant reduction of disability at 90 and 180 days vs. control (*p* = 0.01). This evidence was also confirmed using the AUC calculation, wherein the difference between the global change and the control group was in favor of the merged treated groups (Bayes Factor_1 − 0_ =1.44, *p* = 0.046). Regarding effect size, a mRS mean difference of 2 was associated with a *Hedges' g* equivalent to 0.821 (95% CI: 0.13–1.50) (Figure 5A). Survival assessment showed a significant reduction of mortality risk in the merged treated groups against Group III (*p* = 0.024) with a hazard ratio (HR) of 0.21 (95% CI: 0.06–0.82) at the end of the study (Figure 5B). Additionally, mRS of merged treated groups at 180 days positively correlated to time to treatment (r=0.55, 95% CI: 0.12–0.80, *p* = 0.01).

#### 3.5.1 Utility-weighted mRS

Adopting a patient-centered approach by using utility-weighted mRS, a higher utility mean was demonstrated for merged treated groups when compared to Group III. This effect was evident at 3 months and continued until the last assessment (Table 5).

**Table 5:** Measures of effect size using utility-weight analysis of the mRS.

**Groups**	**Days**	**Utility (Mean ±SD)**	***p*-value**	**Utility mean difference**	**Utility mean difference** ** 95% CI**	**g*_*Hedge*_***	**g*_*Hedge*_* 95% CI**
I+II (*n =* 20)	90	0.73 ± 0.35	0.012^*^	0.26	2.49 × 10^−5^ to 0.58	0.82^£^	0.13–1.52
III (*n =* 16)		0.43 ± 0.38					
I+II (*n =* 20)	180	0.75 ± 0.35	0.011^*^	0.25	3.56 × 10^−5^ to 0.67	0.85^£^	0.16–1.54
III (*n =* 16)		0.42 ± 0.43					

Mean differences were calculated with respect to Group III. ^*^indicates significant differences. ^£^indicates a strong effect (>0.8), according to Chhatbar et al. (50).

## 4 Discussion

The phrase “combination therapy” for stroke usually refers to the use of recanalization approaches along with other therapeutics (13). Few published studies have combined pure neuroprotective drugs under the combined therapy concept (2). Indeed, generally, the use of agents with clear neuroprotective effects appears under the expression of “adjuvant therapy” (53). Our proposal, based on the combination of two pharmacological components with neuroprotective properties, represents a “combination therapy” that does not target the re-opening of the occluded vessel but the protection of remaining viable cells and the strengthening of endogenous regenerative and plasticity mechanisms that might enhance functional recovery. The promising results of this trial showed that EGF+GHRP6 combined therapy is safe. Although not powered for efficacy, this study revealed a significantly better outcome for EGF+GHRP6-treated patients relative to the clinical endpoints of stroke.

The Courage study population had typical risk factors for stroke (54), including older participants, high body mass index, and hypertension. The groups were well balanced with respect to these risk factors and admission stroke scores (Table 1, Figure 2). This baseline balance guarantees the reliability of the results (55).

In this study, the positive and significant correlation between time to treatment and mRS suggests that earlier intervention with EGF+GHRP6 may decrease disability 6 months after the onset of symptoms. This finding is also consistent with our pre-clinical studies (19). Although the immediate intervention is likely to enhance the therapeutic effects, we anticipate that components of this combination therapy will also target later secondary events, promoting survival of the neurovascular unit and activating endogenous neuroprotective pathways, which would predict a longer-lasting effect (56, 57).

The Courage trial based on intravenous delivery of EGF+GHRP6 for acute ischemic stroke was successful in terms of the prespecified primary endpoint concerning safety (Tables 2, 3), confirmed by less than 30% of serious AEs with a causal relationship to treatment, as stated in the hypothesis. Although some AEs, known to be elicited by EGF and GHRP6, were reported, the lack of statistical significance of the relative risks associated with total and serious AEs (Table 2) supports the expected safety profile for this therapeutic approach. At the end of the study, the ethical committees and the independent data monitoring committee issued a favorable assessment in terms of the safety of the therapeutic combination under investigation, endorsing the continuity of the clinical development of the project.

The association between increased survival and combined therapy detected in the prespecified analyses (Figure 4A) is further demonstrated when the two treatment groups are merged (Figure 5B). The hazard ratio indicates a 79% decreased risk of death in patients who received the combined therapy. The survival outcome of this study was consistent with the robust preclinical evidence obtained in animal models of acute focal and global brain ischemia (17, 18). This suggests local and systemic cytoprotection induced by EGF+GHRP6 that eventually preserves organs and systems vulnerable to dysautonomies typical of stroke (58–60).

The additional beneficial effect on functional assessments at 90 and 180 days, as evidenced by dichotomous (Figure 3) and ordinal analyses of merged treated groups (Figure 5A), further supports the likely therapeutic effects of this combination therapy.

Considering that examination of the full range of scores has statistical superiority vs. dichotomous analysis (61), ordinal approaches were also performed along with their associated effect size (mean differences and *Hedges' g*) (Table 4). Our results were consistent across dichotomous and ordinal approaches. The inclusion of effect size analysis not only implies compliance with the CONSORT guidelines (35) but also overcomes the limitations of sample size (62). The moderate to strong effect size of EGF+GHRP6 in the reduction of both stroke severity and resulting disability in treated patients provides additional evidence in favor of this therapeutic approach. Furthermore, the effect size of the mRS at 90 days, which is maintained until 180 days, confirms that the ordinal form of the 3-month mRS correlates better with long-term outcomes (63).

Taking into account that a statistically significant difference is not necessarily translated into a meaningful difference for both patients and clinicians, minimal clinically important differences (MCID) are a better point of reference than the traditional use of statistical significance (64). MCIDs for the NIHSS and mRS scales are 2 points (65) and 1 point (66), respectively. For the Barthel index, an MCID of 1.85 has been reported for a 15-point Likert-type scale (64). By analogy, this may be translated into an MCID of 12.3 change score for the 100-point Barthel index. In this study, the mean differences of NIHSS, Barthel index, and mRS exceeded their respective MCID in the subacute and chronic post-stroke phases (Table 4), showing enhanced outcomes in those patients receiving EGF+GHRP6 therapy. These significant score changes for the three assessment tools are an important finding of this trial as evidence of a real impact on patient perception beyond possible measurement errors (64). The largest MCIDs have such strong evidence that they have been considered the defining criteria for superior clinical trials (67).

In stroke, as in other disabling diseases, the most widely accepted patient-centered outcome measure is utility in terms of the desirability of a specific health outcome to the patient (68). In this study, the effect size of EGF+GHRP6 based on utility-weighted (UW) mRS was relatively large at 3 months and maintained until the last evaluation of the trial (6 months). The analysis of UW-mRS has been a strong recommendation of the Stroke Therapy Academic Industry Roundtable (STAIR) in stroke clinical trials (52), to rate disability outcomes with greater accuracy than unweighted approaches to mRS analysis (69).

An additional feature of UW-mRS is the ability to generate QALYs gained or lost by an intervention or treatment (52). If the current data were reproduced in a study designed for efficacy purposes, our therapeutic approach would confer 0.06 QALYs for every 6 months of survival. The concurrent inclusion of clinically interpretable and reliable mRS along with a contextually appropriate health utility scale allowed us to characterize favorable and meaningful differences in the patient's quality of life (70).

The EGF+GHRP6 combined therapy for stroke was designed to activate a cascade of endogenous neuroprotection mechanisms, including regeneration of adult neural stem cells and repair in the penumbra zone (26–31, 57, 71, 72). The activation of these pathways could explain the clinical (neurological, functional, and survival) outcomes evidenced in this study, which had been previously demonstrated in stroke animal models (17–19). The events induced by both active agents in this combined therapy have a scope that transcends beyond the acute phase of brain infarction (57, 71, 73, 74). The control of infarct core expansion also systemically influences the autonomous functioning of cardiovascular systems, usually affected by stroke (58–60).

The study was limited by a low sample size and a non-blinded design. This could lead to overestimated treatment effects from subjective measures (75). Another important limitation of this study was that imaging could only be used for diagnosis, specifically to rule out the presence of hemorrhagic stroke, and not as an evolutionary parameter together with the clinical outcomes. Additionally, standard therapy did not include thrombolysis, as this treatment is not currently widely available in Cuba for stroke therapy.

Furthermore, the exclusion of patients with NIHSS < 5 and > 20 rendered a more clinically homogeneous population, although the inclusion of only moderately affected patients (median stroke severity of 9) could reduce the discriminative power and the possibility of showing a beneficial effect of any therapy under study, as noted by Ehrenreich et al. (46).

In conclusion, EGF+GHRP6-based therapy is biologically plausible as a therapeutic intervention, given that both serum levels of EGF and insulin-like growth factor-1 (IGF-1) (as the main effect induced by GHRP6) were substantially lower in stroke patients and are determinant factors of ischemic stroke outcomes (76, 77). Thus, this combined therapy could also be considered as a replacement therapy.

For the purposes of stroke interventions, the neuroprotective properties of EGF and GHRP6, in addition to the molecular and cellular events they trigger, suggest a long-term effect, possibly generating trophic effects both in compromised tissue of the ischemic penumbra and in cells with regenerative potential. Intervention with EGF+GHRP6 demonstrates that the treatment is safe and provides encouraging evidence of therapeutic effects. These data provide support for the design of a large pivotal trial, ideally in combination with thrombolytic therapy.

## 5 Conclusion

Effective neuroprotective therapies remain an unmet clinical and social need, especially for patients not tributary to recanalization options. Advantageously, neuroprotective therapies with a high safety profile could always be combined with recanalization alternatives and even be used in subacute or chronic periods along with and after rehabilitation therapies.

This trial has demonstrated the safety of this combination therapy, and although underpowered, the data demonstrate robust evidence of therapeutic effects, both with respect to functional outcome and survival.

## Data availability statement

The raw data supporting the conclusions of this article will be made available by the authors, without undue reservation.

## Ethics statement

The studies involving humans were approved by the Ethic and Review Committee and /or institutional review board of every hospital or clinical sites as follow: Dr. Antonio Luaces Iraola Castro Hospital, Camilo Cienfuegos Hospital, Carlos Manuel de Céspedes Hospital, Celia Sánchez Manduley Hospital, Mártires del 4 de Abril Hospital, Juan Bruno Zayas Hospital, Agostinho Neto Hospital, Dr. Ernesto Guevara Hospital, and Faustino Pérez Hospital. The studies were conducted in accordance with the local legislation and institutional requirements. The participants provided their written informed consent to participate in this study.

## Author contributions

FH-B: Conceptualization, Formal analysis, Methodology, Writing – review & editing, Project administration, Supervision. DE-G: Data curation, Investigation, Writing – review & editing. JG-R: Data curation, Investigation, Writing – review & editing. YM-B: Data curation, Resources, Supervision, Writing – review & editing. KC-Á: Data curation, Resources, Supervision, Writing – review & editing. MG-C: Data curation, Investigation, Writing – review & editing. MG-R: Data curation, Investigation, Writing – review & editing. AC-J: Data curation, Investigation, Writing – review & editing. YF-G: Data curation, Investigation, Writing – review & editing. YP-C: Data curation, Investigation, Writing – review & editing. CV-S: Formal analysis, Writing – review & editing. VM-G: Project administration, Resources, Writing – review & editing. HP-S: Conceptualization, Methodology, Writing – review & editing. NS-M: Conceptualization, Formal analysis, Visualization, Writing – original draft, Writing – review & editing. GG-N: Conceptualization, Formal analysis, Resources, Writing – review & editing. DG-d-B-H: Conceptualization, Formal analysis, Methodology, Visualization, Writing – original draft, Writing – review & editing.

## COURAGE research group

Jorge L. Alonso Freire, Liliana Renté Cantillo, Rogelio Creagh Bandera, Ilietys Borroto Carpio, Héctor Santana Milian, Amaury del Puerto Cruz, Javier J. García Sacarías, Milagro Rodríguez Rosell, José L. Rodríguez Reynoso, Elio Llerena Rodríguez, Grettel Melo Suárez, Elizeth García Iglesias, Marel Alonso Abad, Laurina Hernández Turiño, Idania Baladrón Castrillo, María M. Ríos Cabrera, Ketty Cruz Chirino, Marisol Cruz Díaz, Misleydis Camejo Alemán, Yelién Martín Fadragas, Lourdes M. Basanta Marrero, Milagro Rodríguez González, Ahmed Ruiz Moré, Alina M. Alonso Ruiz, Nelson Rojas Sierra, Anisbel Rodríguez López, Erisdania Esquivel Ferrer, Carmen Remón Chávez, Yoska Castro Jeréz, Virginia Robledo Cabrera, Odalis Mora Barbán, Silvia Vázquez Labrada, Danay Duharte Sarmiñón, Yaritza Alvarado Rey, Adanais González Flores, Ledis Garbey Delás, Yirsa Luna Negret, Tamara Hudson Megret, Mayda Cisnero Rubalcaba, Adelina S. Ferrer Fernández, Idalmis Quevedo Palomo, Yadileydi Elias Oquendo, Dimitri Dueñas Valdivia, Alexis Suárez Quesada, Camelia Valhuerdi, Enrique Rodríguez, Cecilia Sayoux, Niurka Arteaga, Amelia González, Olaida Valdivia, Marta Pérez, Herminia Rodríguez, Indira Brito, Evelin Rangel, Maylín Ortiz, Yamila Espinosa, Yaila Ramírez, Marelis Pantoja, Caridad Guerra, Ana Santana, Salustina Sánchez, Carlos Casas, Norma Mora, Yoelis Copello, Melitza Pupo, Karen Alí, María C. López, Pilar Laborí, Elizabeth Lobaina, Jorge R. Góngora, Yomaidis Araujo, José A. Martínez, Raúl Domínguez, Mae Pupo, Tatiana Marañón, Yadira Hidalgo Boza, Tania González López, and Silvia Barcelona Pérez.

## References

[1] Roy-O'Reilly M McCullough LD. Sex differences in stroke: the contribution of coagulation. Exp Neurol. (2014) 259:16–27. 10.1016/j.expneurol.2014.02.011 24560819 PMC4127336

[2] Paul S Candelario-Jalil E. Emerging neuroprotective strategies for the treatment of ischemic stroke: an overview of clinical and preclinical studies. Exp Neurol. (2021) 335:113518. 10.1016/j.expneurol.2020.113518 33144066 PMC7869696

[3] Ramos-Araque ME Siegler JE Ribo M Requena M Lopez C de Lera M. Stroke etiologies in patients with COVID-19: the SVIN COVID-19 multinational registry. BMC Neurol. (2021) 21:43. 10.1186/s12883-021-02075-1 33514335 PMC7846488

[4] Aguiar de Sousa D von Martial R Abilleira S Gattringer T Kobayashi A Gallofré M. Access to and delivery of acute ischaemic stroke treatments: a survey of national scientific societies and stroke experts in 44 European countries. Eur Stroke J. (2019) 4:13–28. 10.1177/2396987318786023 31165091 PMC6533860

[5] Yang P Zhang Y Zhang L Zhang Y Treurniet KM Chen W. Endovascular thrombectomy with or without intravenous alteplase in acute stroke. New Engl J Med. (2020) 382:1981–93. 10.1056/NEJMoa2001123 32374959

[6] Neuhaus AA Couch Y Hadley G Buchan AM. Neuroprotection in stroke: the importance of collaboration and reproducibility. Brain. (2017) 140:2079–92. 10.1093/brain/awx126 28641383

[7] Saini V Guada L Yavagal DR. Global epidemiology of stroke and access to acute ischemic stroke interventions. Neurology. (2021) 97:S6–S16. 10.1212/WNL.0000000000012781 34785599

[8] Zhao L-R Willing A. Enhancing endogenous capacity to repair a stroke-damaged brain: an evolving field for stroke research. Prog Neurobiol. (2018) 163:5–26. 10.1016/j.pneurobio.2018.01.004 29476785 PMC6075953

[9] Konduri P van Voorst H Bucker A van Kranendonk K Boers A Treurniet K. Posttreatment ischemic lesion evolution is associated with reduced favorable functional outcome in patients with stroke. Stroke. (2021) 52:3523–31. 10.1161/STROKEAHA.120.032331 34289708

[10] Xiong XY Liu L Yang QW. Refocusing neuroprotection in cerebral reperfusion era: new challenges and strategies. Front Neurol. (2018) 9:249. 10.3389/fneur.2018.00249 29740385 PMC5926527

[11] Wang L Xiong X Zhang L Shen J. Neurovascular unit: a critical role in ischemic stroke. CNS Neurosci Ther. (2021) 27:7–16. 10.1111/cns.13561 33389780 PMC7804897

[12] del Zoppo GJ. The neurovascular unit in the setting of stroke. J Intern Med. (2010) 267:156–71. 10.1111/j.1365-2796.2009.02199.x 20175864 PMC3001328

[13] Chamorro Á Lo EH Renú A van Leyen K Lyden PD. The future of neuroprotection in stroke. J Neurol Neurosurg Psychiatr. (2021) 92:129–35. 10.1136/jnnp-2020-324283 33148815

[14] Marmolejo-Martínez-Artesero S Casas C Romeo-Guitart D. Endogenous mechanisms of neuroprotection: to boost or not to be. Cells. (2021) 10:370. 10.3390/cells10020370 33578870 PMC7916582

[15] del Barco DG Montero E Coro-Antich RM Brown E Suarez-Alba J Lopez L. Coadministration of epidermal growth factor and growth hormone releasing peptide-6 improves clinical recovery in experimental autoimmune encephalitis. Restor Neurol Neurosci. (2011) 29: 243–52. 10.3233/RNN-2011-0595 21697595

[16] Del Barco DG Perez-Saad H Rodriguez V Marin J Falcon V Martin J. Therapeutic effect of the combined use of growth hormone releasing peptide-6 and epidermal growth factor in an axonopathy model. Neurotox Res. (2011) 19:195–209. 10.1007/s12640-010-9160-8 20169434

[17] Garcia Del Barco-Herrera D Martinez NS Coro-Antich RM Machado JM Alba JS Salgueiro SR. Epidermal growth factor and growth hormone-releasing peptide-6: combined therapeutic approach in experimental stroke. Restor Neurol Neurosci. (2013) 31:213–23. 10.3233/RNN-120262 23314006

[18] Subiros N Perez-Saad H Aldana L Gibson CL Borgnakke WS Garcia-Del-Barco D. Neuroprotective effect of epidermal growth factor plus growth hormone-releasing peptide-6 resembles hypothermia in experimental stroke. Neurol Res. (2016) 38:950–8. 10.1080/01616412.2016.1235249 27665924

[19] Subiros N Perez-Saad HM Berlanga JA Aldana L Garcia-Illera G Gibson CL. Assessment of dose-effect and therapeutic time window in preclinical studies of rhEGF and GHRP-6 coadministration for stroke therapy. Neurol Res. (2016) 38:187–95. 10.1179/1743132815Y.0000000089 26311576

[20] Yang S Jin H Zhao ZG. Epidermal growth factor treatment has protective effects on the integrity of the blood-brain barrier against cerebral ischemia injury in bEnd3 cells. Exp Ther Med. (2019) 17:2397–402. 10.3892/etm.2019.7186 30867725 PMC6396002

[21] Pan W Kastin AJ. Entry of EGF into brain is rapid and saturable. Peptides. (1999) 20:1091–8. 10.1016/S0196-9781(99)00094-7 10499427

[22] Kumarnsit E Johnstone LE Leng G. Actions of neuropeptide Y and growth hormone secretagogues in the arcuate nucleus and ventromedial hypothalamic nucleus. Eur J Neurosci. (2003) 17:937–44. 10.1046/j.1460-9568.2003.02521.x 12653970

[23] Dickson SL Leng G Dyball REJ Smith RG. Central actions of peptide and non-peptide growth hormone secretagogues in the rat. Neuroendocrinology. (1995) 61:36–43. 10.1159/000126825 7731496

[24] Guan X-M Yu H Palyha OC McKee KK Feighner SD Sirinathsinghji DJ. Distribution of mRNA encoding the growth hormone secretagogue receptor in brain and peripheral tissues. Mol brain Res. (1997) 48:23–9. 10.1016/S0169-328X(97)00071-5 9379845

[25] Novak U Walker F Kaye A. Expression of EGFR-family proteins in the brain: role in development, health and disease. J Clin Neurosci. (2001) 8:106–11. 10.1054/jocn.2000.0799 11243764

[26] Niidome T Morimoto N Iijima S Akaike A Kihara T Sugimoto H. Mechanisms of cell death of neural progenitor cells caused by trophic support deprivation. Eur J Pharmacol. (2006) 548:1–8. 10.1016/j.ejphar.2006.07.052 16965769

[27] Delgado-Rubin A Chowen JA Argente J Frago LM. Growth hormone-releasing peptide 6 protection of hypothalamic neurons from glutamate excitotoxicity is caspase independent and not mediated by insulin-like growth factor I. Eur J Neurosci. (2009) 29:2115–24. 10.1111/j.1460-9568.2009.06770.x 19490089

[28] Casper D Blum M. Epidermal growth factor and basic fibroblast growth factor protect dopaminergic neurons from glutamate toxicity in culture. J Neurochem. (1995) 65:1016–26. 10.1046/j.1471-4159.1995.65031016.x 7643081

[29] Delgado-Rubín de Célix A Chowen JA Argente J Frago LM. Growth hormone releasing peptide-6 acts as a survival factor in glutamate-induced excitotoxicity. J Neurochem. (2006) 99:839–49. 10.1111/j.1471-4159.2006.04122.x 17076656

[30] Scalabrino G. Epidermal growth factor in the CNS: a beguiling journey from integrated cell biology to multiple sclerosis. An extensive translational overview. Cell Mol Neurobiolgy. (2020) 21:1–26. 10.1007/s10571-020-00989-x 33151415 PMC8942922

[31] Frago L. M. Chowen J. A. Basic Physiology of the Growth Hormone/Insulin-Like Growth Factor Axis. In: Varela-Nieto I Chowen J, editors. The Growth Hormone/Insulin-Like Growth Factor Axis During Development. Boston, MA: Springer (2005). p. 1–25. 10.1007/0-387-26274-1_1 16370134

[32] Cabrales A Gil J Fernandez E Valenzuela C Hernandez F Garcia I. Pharmacokinetic study of growth hormone-releasing peptide 6 (GHRP-6) in nine male healthy volunteers. Eur J Pharm Sci. (2013) 48:40–6. 10.1016/j.ejps.2012.10.006 23099431

[33] Palomino A Hernandez-Bernal F Haedo W Franco S Mas J Fernández J. A multicenter, randomized, double-blind clinical trial examining the effect of oral human recombinant epidermal growth factor on the healing of duodenal ulcers. Scand J Gastroenterol. (2000) 35:1016–22. 10.1080/003655200451126 11099053

[34] Ioannidis JP Evans SJ Gøtzsche PC O'Neill RT Altman DG Schulz K. Better reporting of harms in randomized trials: an extension of the CONSORT statement. Annal Int Med. (2004) 141:781–8. 10.7326/0003-4819-141-10-200411160-00009 15545678

[35] Moher D Hopewell S Schulz KF Montori V Gøtzsche PC Devereaux PJ. CONSORT 2010 Explanation and Elaboration: updated guidelines for reporting parallel group randomised trials. BMJ. (2010) 340:c869. 10.1136/bmj.c869 20332511 PMC2844943

[36] CECMED. Center for state control on the quality of medicines. Good clinical practices in Cuba. MINSAP Resol. (2000) 165:48.

[37] Dixon JR. The international conference on harmonization good clinical practice guideline. Q Assurance. (1999) 6:65–74. 10.1080/105294199277860 10386329

[38] World Medical Association. World Medical Association Declaration of Helsinki: ethical principles for medical research involving human subjects. JAMA. (2013) 310:2191–4. 10.1001/jama.2013.281053 24141714

[39] Carter RE Woolson RF. Statistical design considerations for pilot studies transitioning therapies from the bench to the bedside. J Transl Med. (2004) 2:1–3. 10.1186/1479-5876-2-37 15511289 PMC527875

[40] Buergo-Zuaznábar MA Fernández-Concepción O Pérez-Nellar J Lara-Fernández G Maya-Entenza C Pando-Cabrera A. Guías de práctica clínica para las enfermedades cerebrovasculares. Medisur. (2007) 5:20. Available online at: https://www.redalyc.org/articulo.oa?id=180020185002

[41] Sharma V McNeill JH. To scale or not to scale: the principles of dose extrapolation. Br J Pharmacol. (2009) 157:907–21. 10.1111/j.1476-5381.2009.00267.x 19508398 PMC2737649

[42] Naranjo C. Shear N. Busto U. Adverse drug reactions. In: Kalant H Roschlau W, editors. Principles of Medical Pharmacology, 6th Edn. New York, NY: Oxford University Press (1998). p. 791–800.

[43] Fernández-Montequín JI Betancourt BY Leyva-Gonzalez G Mola EL Galán-Naranjo K Ramírez-Navas M. Intralesional administration of epidermal growth factor-based formulation (Heberprot-P) in chronic diabetic foot ulcer: treatment up to complete wound closure. Int Wound J. (2009) 6:67–72. 10.1111/j.1742-481X.2008.00561.x 19291119 PMC7951202

[44] Spilker J Kongable G Barch C Braimah J Bratina P Daley S. Using the NIH Stroke Scale to assess stroke patients. J Neurosci Nurs. (1997) 29:384–93. 10.1097/01376517-199712000-00008 9479660

[45] Lai S-M Duncan PW. Stroke recovery profile and the Modified Rankin assessment. Neuroepidemiology. (2001) 20:26–30. 10.1159/000054754 11174042

[46] Ehrenreich H Hasselblatt M Dembowski C Cepek L Lewczuk P Stiefel M. Erythropoietin therapy for acute stroke is both safe and beneficial. Mol Med. (2002) 8:495–505. 10.1007/BF03402029 12435860 PMC2040012

[47] Thall PF Estey EH. A Bayesian strategy for screening cancer treatments prior to phase II clinical evaluation. Stat Med. (1993) 12:1197–211. 10.1002/sim.4780121303 8210822

[48] Johnson VE. Bayes factors based on test statistics. J Royal Stat Soc Series B. (2005) 67:689–701. 10.1111/j.1467-9868.2005.00521.x

[49] Lenhard W Lenhard A. Computation of effect sizes. Psychometrica. (2016). 10.13140/RG.2.2.17823.92329

[50] Chhatbar PY Ramakrishnan V Kautz S George MS Adams RJ Feng W. Transcranial direct current stimulation post-stroke upper extremity motor recovery studies exhibit a dose–response relationship. Brain Stimul. (2016) 9:16–26. 10.1016/j.brs.2015.09.002 26433609 PMC4724265

[51] Chaisinanunkul N Adeoye O Lewis R Grotta J Broderick J Jovin T. Adopting a patient-centered approach to primary outcome analysis of acute stroke trials using a utility-weighted modified rankin scale. Stroke. (2015) 46:2238–43. 10.1161/STROKEAHA.114.008547 26138130 PMC4519373

[52] Broderick JP Adeoye O Elm J. Evolution of the modified Rankin scale and its use in future stroke trials. Stroke. (2017) 48:2007–12. 10.1161/STROKEAHA.117.017866 28626052 PMC5552200

[53] Zhang L Zhang ZG Chopp M. The neurovascular unit and combination treatment strategies for stroke. Trends Pharmacol Sci. (2012) 33:415–22. 10.1016/j.tips.2012.04.006 22595494 PMC3407317

[54] Dearborn J. L. Gottesman R. F. Hypertension: The Major Risk Factor for Stroke. In: Seshadri S Debette S, editors. Risk Factors for Cerebrovascular Disease and Stroke. Oxford: Oxford University Press (2016), p. 155–79.

[55] Alper BS Foster G Thabane L Rae-Grant A Malone-Moses M Manheimer E. Thrombolysis with alteplase 3-45 hours after acute ischaemic stroke: trial reanalysis adjusted for baseline imbalances. BMJ Evid Based Med. (2020) 25:168–71. 10.1136/bmjebm-2020-111386 PMC7548536 32430395

[56] Johansson I Destefanis S Aberg ND Åberg MA Blomgren K Zhu C. Proliferative and protective effects of growth hormone secretagogues on adult rat hippocampal progenitor cells. Endocrinology. (2008) 149:2191–9. 10.1210/en.2007-0733 18218693

[57] Romano R Bucci C. Role of EGFR in the nervous system. Cells. (2020) 9:1887. 10.3390/cells9081887 32806510 PMC7464966

[58] Nayani S Sreedharan SE Namboodiri N Sarma PS Sylaja P. Autonomic dysfunction in first ever ischemic stroke: prevalence, predictors and short term neurovascular outcome. Clin Neurol Neurosurg. (2016) 150:54–8. 10.1016/j.clineuro.2016.08.022 27588371

[59] Jimenez-Ruiz A Racosta JM Kimpinski K Hilz MJ Sposato LA. Cardiovascular autonomic dysfunction after stroke. Neurol Sci. (2021) 42:1751–8. 10.1007/s10072-021-05128-y 33687612

[60] Constantinescu V Arsenescu-Georgescu C Matei D Moscalu M Corciova C Cuciureanu D. Heart rate variability analysis and cardiac dysautonomia in ischemic stroke patients. Clin Neurol Neurosurg. (2019) 186:105528. 10.1016/j.clineuro.2019.105528 31574361

[61] Ganesh A Luengo-Fernandez R Pendlebury ST Rothwell PM Study OV. Weights for ordinal analyses of the modified Rankin Scale in stroke trials: a population-based cohort study. EClinicalMedicine. (2020) 23:100415. 10.1016/j.eclinm.2020.100415 32577611 PMC7300241

[62] Sullivan GM Feinn R. Using effect size—or why the P value is not enough. J Grad Med Educ. (2012) 4:279–82. 10.4300/JGME-D-12-00156.1 23997866 PMC3444174

[63] Ganesh A Luengo-Fernandez R Wharton RM Rothwell PM. Ordinal vs dichotomous analyses of modified Rankin Scale, 5-year outcome, and cost of stroke. Neurology. (2018) 91:e1951–e60. 10.1212/WNL.0000000000006554 30341155 PMC6260198

[64] Hsieh Y-W Wang C-H Wu S-C Chen P-C Sheu C-F Hsieh C-L. Establishing the minimal clinically important difference of the Barthel Index in stroke patients. Neurorehabil Neural Repair. (2007) 21:233–8. 10.1177/1545968306294729 17351082

[65] Ma Y Deng K Liu J Ma B Mei F Hui W. The add-on effects of Danhong injection among patients with ischemic stroke receiving Western medicines: A systematic review and meta-analysis. Front Pharmacol. (2022) 13:937639. 10.3389/fphar.2022.937369 36081951 PMC9445550

[66] Narayan Arya K Verma R Garg R. Estimating the minimal clinically important difference of an upper extremity recovery measure in subacute stroke patients. Top Stroke Rehabil. (2011) 18:599–610. 10.1310/tsr18s01-599 22120029

[67] Cranston JS Kaplan BD Saver JL. Minimal clinically important difference for safe and simple novel acute ischemic stroke therapies. Stroke. (2017) 48:2946–51. 10.1161/STROKEAHA.117.017496 28931621

[68] Feeny D. A utility approach to the assessment of health-related quality of life. Med Care. (2000) 38:II151–II4. 10.1097/00005650-200009002-00022 10982100

[69] Tokunboh I Sung EM Chatfield F Gaines N Nour M Starkman S. Improving Visualization Methods of Utility-Weighted Disability Outcomes for Stroke Trials. Frontiers in Neurology (2022) 998. 10.3389/fneur.2022.875350 PMC9136165 35645952

[70] Rebchuk A.D., O'Neill Z.R., Szefer E.K., Hill M.D., Field T.S. Health utility weighting of the modified Rankin scale: a systematic review and meta-analysis. JAMA network open (2020) 3: e203767-e. 10.1001/jamanetworkopen.2020.3767 PMC7191324 32347948

[71] Chan SJ Love C Spector M Cool SM Nurcombe V Lo EH. Endogenous regeneration: Engineering growth factors for stroke. Neurochem Int. (2017) 107:57–65. 10.1016/j.neuint.2017.03.024 28411103

[72] Aguirre A Dupree JL Mangin J Gallo V. A functional role for EGFR signaling in myelination and remyelination. Nat Neurosci. (2007) 10:990–1002. 10.1038/nn1938 17618276

[73] Xin W-Q Wei W Pan Y-L Cui B-L Yang X-Y Bähr M. Modulating poststroke inflammatory mechanisms: novel aspects of mesenchymal stem cells, extracellular vesicles and microglia. World J Stem Cells. (2021) 13:1030. 10.4252/wjsc.v13.i8.1030 34567423 PMC8422926

[74] Dhir N Medhi B Prakash A Goyal MK Modi M Mohindra S. Pre-clinical to clinical translational failures and current status of clinical trials in stroke therapy: A brief review. Curr Neuropharmacol. (2020) 18:596–612. 10.2174/1570159X18666200114160844 31934841 PMC7457423

[75] Hróbjartsson A Emanuelsson F Skou Thomsen AS Hilden J Brorson S. Bias due to lack of patient blinding in clinical trials. A systematic review of trials randomizing patients to blind and nonblind sub-studies. Int J Epidemiol. (2014) 43:1272–83. 10.1093/ije/dyu115 24881045 PMC4258786

[76] Øverberg LT Lugg EF Gaarder M Langhammer B Thommessen B Rønning OM. Plasma levels of BDNF and EGF are reduced in acute stroke patients. Heliyon. (2022) 12:e09661. 10.1016/j.heliyon.2022.e09661 35756121 PMC9218156

[77] Hayes CA Valcarcel-Ares MN Ashpole NM. Preclinical and clinical evidence of IGF-1 as a prognostic marker and acute intervention with ischemic stroke. J Cereb Blood Flow Metab. (2021) 41:2475–91. 10.1177/0271678X211000894 33757314 PMC8504958

